# WAFF: A Synergetic Face Forgery Video Detection Method via Weakly Supervised EfficientNet

**DOI:** 10.3390/jimaging12060240

**Published:** 2026-05-29

**Authors:** Zhengzhuo Pan, Bohan Chen, Longxiang Ma, Dawei Jin, Yu Zhou, Yudi Huang

**Affiliations:** 1 School of Statistics and Mathematics, Zhongnan University of Economics and Law, Wuhan 430079, China; panzhengzhuo@stu.zuel.edu.cn; 2 School of Information Engineering, Zhongnan University of Economics and Law, Wuhan 430079, China; 202321130351@stu.zuel.edu.cn (B.C.); 202321130220@stu.zuel.edu.cn (L.M.); jdw@zuel.edu.cn (D.J.); zhouyu_cs@zuel.edu.cn (Y.Z.)

**Keywords:** deepfake detection, face forgery, weakly supervised attention

## Abstract

Deepfake detection has become an essential task for ensuring the authenticity and security of digital media. Although recent approaches have achieved notable progress, most existing detectors still exhibit limited generalization to unseen forgery techniques and remain vulnerable to common perturbations such as compression, noise, and adversarial attacks. To overcome these issues, we propose Weakly Supervised EfficientNet Augmented Face Forgery Detector (WAFF), a novel framework that integrates fine-grained per-frame analysis with adaptive video-level fusion. Specifically, WAFF integrates WSEffiNet, an EfficientNet-B3-based backbone enhanced with a Weakly Supervised Data Augmentation Network (WS-DAN). This design generates attention maps to emphasize subtle facial forgery artifacts while encouraging complementary local–global feature learning. At the video level, WAFF incorporates a multi-strategy fusion scheme that combines fake-frame counting, confidence averaging, and attention-guided voting to strike a balance between sensitivity and stability. Extensive experiments on FaceForensics++, Celeb-DF v2, DFD, DFDC, and FFIW-10K demonstrate that WAFF can achieve state-of-the-art performance under both high- and low-quality compression, while also enhancing cross-dataset generalization.

## 1. Introduction

Since the emergence of deep generative models, deepfake technology has evolved rapidly. It has shifted from a niche research topic to widely accessible tools, enabling the easy creation of highly convincing facial manipulations that often escape casual observation. Recent advancements in generative adversarial networks (GANs), variational autoencoders (VAEs), and attention-based architectures have enabled seamless modifications of facial identity, expressions, lip movements, and other features [1,2]. Although these tools offer opportunities in creative domains such as film post-production and virtual try-on, their accessibility has also fueled the rise of digital forgeries [3]. This trend raises serious concerns about trust, privacy, and the integrity of public discourse. For instance, individuals with little legal awareness can create fake videos of public figures to spread misinformation or attempt extortion. Such incidents prompt urgent calls for effective forensic detection methods from regulators and platform operators [4,5,6].

Conventionally, deepfake detection is formulated as a binary classification problem [7,8]. To identify whether a segment of video is forged or not, researchers have adopted two primary methodologies: The first focuses on intra-frame analysis, scrutinizing individual video frames for visual inconsistencies, such as frequency-domain artifacts resulting from upsampling, color mismatches at facial boundaries, or subtle semantic discrepancies between manipulated areas and their surroundings [9,10]. Although these methods often achieve high accuracy against known manipulation techniques, they struggle with novel or heavily compressed forgeries [11]. Moreover, their dependence on standard CNN feature extractors limits their ability to detect subtle, localized artifacts such as those around the corners of the eyes or the boundaries of the lips, which reveal a synthetic origin [7,12]. The second type of approach utilizes temporal cues across multiple frames, examining features like eye-blink frequency, coherence of optical flow, or the dynamics of deep features learned by recurrent networks to identify unnatural motion patterns [13]. Although temporal methods generally demonstrate a higher resilience to compression artifacts, they require longer video inputs and entail considerable computational costs. These factors may limit their practicality for real-time applications or large-scale deployments [14,15]. Furthermore, both intra-frame and temporal techniques often exhibit limited generalization when confronted with previously unseen forgery algorithms.

Recent research has delved deeper into these limitations, revealing that current detectors struggle significantly with cross-dataset evaluations and real-world corruptions [16,17]. One category of approaches leverages generative adversarial networks (GANs) with dual generators—blend-based for adaptive masking and blending, and transfer-based for style mixing—to create challenging synthetic forgeries that bridge in-dataset and cross-dataset gaps by simulating divergent synthetic patterns, such as those from techniques like DF-VAE or NeuralTextures [18]. These GAN-based methods often incorporate collaborative spatial-frequency discriminators to detect artifacts while filtering out perturbations like blur, noise, or adversarial attacks, thereby enhancing overall robustness. Another class of algorithms emphasizes inconsistency learning through advanced image blending, introducing bi-level inconsistencies—extrinsic between real and pseudo-forged regions, and inherent between real and manipulated areas—to better replicate common forgery clues and improve generalization across diverse datasets, as evidenced by improved metrics on benchmarks like DFDC and Celeb-DF [19,20]. These developments underscore the essential requirement for detection frameworks that not only adapt to unseen forgeries but also withstand various real-world distortions.

Despite promising progress, two inevitable challenges remain in practical deployment: (1) the limited generalization of detectors to unseen forgery generation techniques and cross-dataset scenarios, where texture patterns may differ drastically; and (2) the lack of robustness to perturbations such as compression, noise, and adversarial attacks, which can easily corrupt discriminative cues and lead to misclassification.

Addressing the limitations of existing approaches, this paper proposes WAFF as a video-level face forgery detection framework, rather than merely as a replacement of an existing backbone. The novelty lies in the task-specific integration of weakly supervised attention learning, complementary local–global augmentation, and calibrated video-level evidence fusion for robust deepfake detection under compression and dataset shift. The significant contributions of this paper are as follows:

(1) We design WSEffiNet, an EfficientNet-B3-based detector enhanced by weakly supervised attention. Instead of requiring pixel-level forgery masks or manually annotated artifact regions, WSEffiNet learns discriminative attention maps from image-level real/fake labels and uses them to guide bilinear attention pooling, attention cropping, and attention dropping.
(2) For video-level classification, WAFF incorporates a calibrated fusion module that integrates fake-frame counting, average confidence scoring, key-frame voting, and attention-guided weighting. This design explicitly balances sensitivity to intermittent forgery cues with stability against isolated false alarms.
(3) Experiments conducted on the public datasets FaceForensics++, Celeb-DF v2, DFD, DFDC, and FFIW-10K show that WAFF generally outperforms state-of-the-art baselines in both in-dataset and cross-dataset settings. The evaluation further analyzes compression robustness, decision-rule behavior, weak versus full supervision, preprocessing sensitivity, and error cases, thereby clarifying both the strengths and the deployment limitations of the proposed framework.

## 2. Related Works

### 2.1. Face Forgery Generation Techniques

The rapid advancement of deep generative models has significantly enhanced face forgery techniques, allowing for the creation of highly realistic yet potentially misleading facial content. These methods can generally be classified into two primary categories: identity swap, which encompasses technologies such as DeepFakes [21], FaceSwap [22,23], and FaceShifter [24]; and expression reenactment, which includes tools like Face2Face [25,26] and NeuralTextures [27,28].

Identity swapping involves replacing an individual’s facial identity while maintaining the target’s expressions and pose. Earlier methods primarily utilized autoencoders or GANs to encode and decode facial features. However, more recent advances in identity swapping have increasingly relied on large pretrained generative models such as StyleGAN. Li et al. [29] proposed FaceSwapper, which employs an identity encoder and an attribute encoder to disentangle identity and non-identity features within the latent representation. This disentanglement enables accurate one-shot face swapping while maintaining the target’s attributes and details, thereby achieving a good trade-off between fidelity and realism. Similarly, Luo et al. [30] introduced StyleFace, which represents identity as a latent variable and integrates an adaptive attribute extractor to preserve target-specific attributes. By incorporating contrastive learning into the StyleGAN latent space, the framework achieves megapixel-level face synthesis with detail preservation and high visual realism.

On the other hand, expression reenactment focuses on animating the target face using expressions, pose, or audio from a source actor. Deng et al. [31] proposed a method that leverages 3D-aware generative radiance manifolds to synthesize high-fidelity, 3D-consistent portraits from monocular images, enabling expression transfer by reconstructing detailed facial geometry and appearance for realistic rendering. Thies et al. [32] developed an audio-driven model that generates realistic lip-sync and head movement in talking-head videos by learning a joint audio–visual embedding, thereby achieving temporal coherence across frames.

Recent studies have analyzed the observable visual and temporal artifacts left by deepfake video generation and their implications for detection. Gong and Li [33] systematically summarized that manipulated facial regions often present irregular facial boundaries, texture distortions, and blending inconsistencies that deviate from natural facial statistics, and they emphasized that such spatial artifacts provide fundamental cues for deepfake detection. Beyond spatial anomalies, deepfake videos have been demonstrated to suffer from pronounced temporal incoherence across consecutive frames. Amin et al. [34] showed that forged videos frequently exhibit abnormal temporal variations in facial appearance and motion consistency, and they validated that modeling temporal coherence significantly improves video-level deepfake detection compared with frame-wise analysis. In addition, recent studies have shown that manipulations often manifest as spatiotemporal inconsistencies rather than purely spatial anomalies, introducing both localized facial artifacts and disrupted temporal coherence across frames, which motivates spatiotemporal and attention-guided detection strategies [35].

### 2.2. Face Forgery Detection Methods

Recent advancements in face forgery detection have evolved from classical binary classification frameworks to techniques prioritizing cross-dataset generalization and robustness against real-world distortions. Early approaches leveraged convolutional neural networks for artifact detection. For example, Rössler et al. [21] introduced an XceptionNet-based detector trained on the FaceForensics++ dataset, leveraging depthwise separable convolutions to discern traces of manipulation within controlled experimental conditions. Li and Lyu [36] proposed DSP-FWA, which identifies facial warping artifacts through optical flow analysis, thereby attaining substantial in-dataset performance by targeting geometric inconsistencies. Likewise, Li et al. [37] presented Face X-ray, which visualizes blending boundaries using a differentiable X-ray representation, facilitating the detection of common forgery pipelines in the absence of method-specific training.

As the limitations of dataset-specific training became apparent, subsequent works began to emphasize cross-dataset generalization. Luo et al. proposed FRDM [38], which mines high-frequency features to capture transferable forgery cues beyond spatial domains. Similarly, Dong et al. introduced CADDM [39], addressing the problem of implicit identity leakage through domain adaptation; while Chen et al. developed SLADD [40], a self-supervised adversarial framework that generates blended samples for robust training. In parallel, frequency-based and transformer-based approaches have also been explored. Zhuang et al. [41] presented UIA-ViT, an inconsistency-aware vision transformer that models patch-level discrepancies globally. More recently, Xu et al. introduced FD-GAN [18], which leverages dual generators and collaborative spatial-frequency discriminators to synthesize diverse forgeries and enhance resistance to compression and adversarial perturbations.

Beyond frame-level analysis, video-based methods incorporate temporal information to exploit motion and consistency cues. Masi et al. [42] proposed a two-branch recurrent network that models both spatial and temporal features, while Zhou et al. [43] introduced LAMP and DAM, which learn local inconsistencies across diverse real-world settings. Hu et al. [44] further developed Finfer, a frame inference model that leverages inter-frame coherence to detect subtle blending artifacts. Although these temporal methods improve resilience to compression and enhance generalization, they often come at the cost of increased computational overhead and limited scalability.

Recent survey- and system-level studies further indicate that practical deepfake detection must be evaluated beyond a single in-dataset accuracy number. Heidari et al. [45] reviewed deep-learning-based deepfake detectors and emphasized persistent challenges in cross-dataset generalization, robustness, and over-reliance on accuracy-only reporting. Raza et al. [46] similarly argued that modern detectors should balance accuracy, computational feasibility, and generalization under distribution shift. In addition, blockchain and federated learning approaches have been explored to address privacy-preserving collaborative detection in realistic deployment scenarios [47]. These studies motivate our emphasis on video-level robustness, failure-case analysis, and deployment-oriented efficiency.

Overall, while existing works have advanced the field from CNN-based classifiers to frequency-based, transformer-based, GAN-based, and video-level models, challenges remain in achieving both high sensitivity to subtle manipulations and robustness under cross-dataset and real-world perturbations.

## 3. Materials and Methods

**Figure 1:**
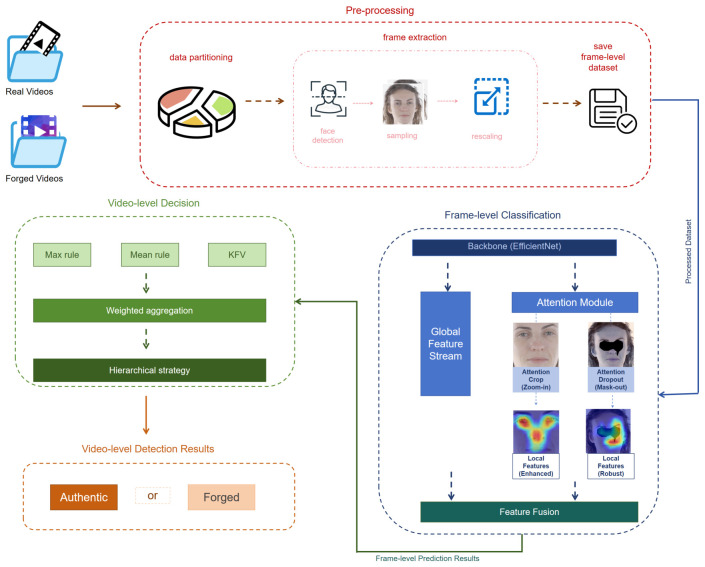
Overall flowchart of the proposed WAFF framework.

This section delineates the proposed WAFF framework, which was developed to address the pressing challenge of robust and scalable video-level forgery detection. As illustrated in Figure 1, WAFF is conceptualized as a three-stage pipeline, furnishing an end-to-end methodology that spans from raw video input to robust video-level inference. Specifically, the framework comprises (1) preprocessing, wherein videos are decomposed into sampled frames and facial regions are localized utilizing InsightFace; (2) frame-level analysis, wherein a novel weakly supervised backbone, designated WSEffiNet (see Section 3.1), is employed to integrate EfficientNet with attention-based augmentation, facilitating the identification of subtle and localized forgery artifacts; and (3) video-level decision rules, wherein frame-level predictions are synthesized via max-based detection, mean-confidence scoring, and key-frame voting to produce the final classification outcome.

The design of WAFF is motivated by the observation that deepfake artifacts are not uniformly distributed across the entire face or consistently present in every frame. Instead, manipulations typically introduce localized visual anomalies in specific facial regions—such as facial boundaries, the mouth, or the nose—and these anomalies may appear intermittently over time. Consequently, effective deepfake detection requires both spatial sensitivity to local artifacts at the frame level and a robust mechanism to aggregate such evidence across frames at the video level.

It is important to note that the weak supervision in WAFF is realized through the design of WSEffiNet, where the WS-DAN module generates attention responses from image-level class labels during training. These responses act as internally produced spatial indicators and are obtained without incorporating region annotations or pixel-level artifact masks. Unlike conventional supervised settings that integrate explicitly defined spatial supervision into the training pipeline, the spatial guidance in WAFF emerges from the model’s own attention mechanism, which derives localized cues implicitly while learning from global labels.

More specifically, each training face crop is supervised only by its binary real/fake label. No manual forgery boundary, manipulated-region mask, landmark-level artifact label, or temporal annotation is used. Given the final EfficientNet-B3 feature tensor, a 1×1 convolution produces *M* attention maps. During training, the attention maps are sampled according to their activation strength: one map guides attention cropping, which zooms into the most discriminative local region, while another guides attention dropping, which suppresses an already salient region and forces the network to discover complementary evidence. In this way, the weak label supervises classification, whereas the attention mechanism supplies self-generated spatial proposals that encourage local–global feature learning.

### 3.1. Architecture Details of WSEffiNet

The proposed WSEffiNet architecture is based on a customized EfficientNet-B3 backbone, shown in Table 1. Compared with the original EfficientNet-B3, we adapt the input resolution to 300×300 for higher spatial fidelity, slightly adjust the number of channels in the later stages (e.g., 136 and 232 channels in Stages 6 and 7), and integrate an attention mechanism before the classification head. These modifications are designed to better capture subtle artifacts in facial forgeries while maintaining efficiency.

Architecturally, WSEffiNet follows a backbone–head design. The EfficientNet-B3 backbone is employed as a feature extractor and preserves its original MBConv and SE structures. The WS-DAN module is integrated at the output of the backbone and operates on the final convolutional feature maps, without altering the intermediate layers. Specifically, WS-DAN takes the last-stage feature tensor as an input to generate multiple attention maps, which are subsequently used for attention pooling, attention-guided cropping, and dropping during training.

The compound scaling principle of EfficientNet governs the design of the backbone:

(1) $$d=\alpha^{\varphi },\;w=\beta^{\varphi },\;r=\gamma^{\varphi },\;\alpha \cdot \beta^{2}\cdot \gamma^{2}\approx 2\;\left(\alpha ,\beta ,\gamma >1\right),$$

where *d*, *w*, and *r* control depth, width, and resolution, respectively. In our case, setting φ=3 corresponds to EfficientNet-B3, which achieves a strong trade-off between accuracy and complexity.

At the core of the backbone is a sequence of Mobile Inverted Bottleneck Convolution (MBConv) blocks, each followed by a Squeeze-and-Excitation (SE) module. An MBConv block first expands the input channels with a 1×1 convolution, applies depthwise convolution over k×k neighborhoods, and projects features back via another 1×1 convolution. The SE module adaptively reweights channels to emphasize informative features. These modules preserve efficiency while improving the representation quality.

After processing an input face image $X\in R^{B\times 3\times 300\times 300}$, the network produces feature maps $F\in R^{B\times 1536\times 10\times 10}$. To focus on discriminative regions, we append a 1×1 convolutional layer that generates *M* attention maps $A\in R^{B\times M\times 10\times 10}$. Instead of uniformly aggregating all regions through global average pooling, we apply bilinear attention pooling (BAP) to selectively weight spatial regions:

(2) $$X_{b,(m,c)}=\frac{1}{H\;W}\sum_{i=1}^{H}\sum_{j=1}^{W}A_{b,m,i,j}\;F_{b,c,i,j},$$

yielding a feature matrix $X\in R^{B\times (M\;C)}$ with tens of thousands of elements.

To stabilize training, each row of *X* is transformed by the sign–sqrt rule and normalized to the unit $\ell_{2}$ sphere:

(3) $$Y=\frac{\mathrm{sign}\left(X\right)\;\sqrt{|X|+\varepsilon }}{∥\mathrm{sign}\left(X\right)\;\sqrt{|X|+\varepsilon }{∥}_{2}},\;\varepsilon =10^{-12}.$$

This enhances discriminability by reducing bursty responses. The classification head is a fully connected layer:

(4) $$\hat{p}=W\;Y+b,$$

producing logits over *K* classes.

To encourage richer representation learning, we adopt a multi-path attention strategy where raw images, attention-guided cropping, and attention-guided dropping are jointly used. This forces the model to focus on both localized fine-grained cues and global context. The classification head is trained with a joint loss that combines cross-entropy and center loss:

(5) $$L=L_{a}+\lambda L_{c},$$

where $L_{a}$ averages cross-entropy over raw, cropped, and dropped paths, while $L_{c}$ enforces intra-class compactness by penalizing deviations from learned class centers.

The integration of compound-scaled MBConv+SE blocks, attention-guided BAP, multi-path attention learning, and the joint objective allows WSEffiNet to maintain efficiency (about 12 M parameters and 1.8 B FLOPs, comparable to EfficientNet-B3) while enhancing sensitivity to subtle deepfake artifacts.

The overall training procedure is summarized in Algorithm 1. Unlike conventional CNN training, our pipeline incorporates attention-guided cropping and dropping to augment feature diversity, as well as a center loss term to enforce compact intra-class representations, making the process tailored to deepfake detection.

### 3.2. Decision Rules

While the proposed WSEffiNet model described in the previous subsection yields frame-level predictions with high discriminative capacity, its practical deployment requires the implementation of a robust mechanism to aggregate and elevate these results to the video level. This step is crucial, since deepfake artifacts may only appear intermittently across frames, and occasional spurious responses could otherwise trigger false alarms.

To address this challenge, the video-level aggregation in WAFF is designed to be evidence-driven rather than purely uniform. Although the decision rules are implemented using simple aggregation operations, they are guided by frame-level prediction confidence and attention responses produced by WSEffiNet. This design allows the aggregation process to account for heterogeneous frame-level signals when forming a video-level decision, rather than assuming equal contribution from all frames.
**Algorithm 1** Training Procedure of the Integrated EfficientNet-B3 and WS-DAN Model**Input:** Training set *D*, model parameters θ, number of epochs *T*, initial learning rate α, batch size *N*, number of attention maps *M***Output:** Updated model parameters θ, feature center matrix *C*  1:Initialize weight parameters  2:Initialize feature center matrix *C*  3:**for** i=1 to *T* **do**  4:      **for** j=1 to *N* **do**  5:            Input image *X* and label *y*  6:            Extract feature map *F* of *X* using EfficientNet-B3  7:            Apply 1×1 convolution on *F* to obtain attention map $A\in R^{B\times M\times H\times W}$  8:            **for** k=1 to *M* **do**  9:                  Compute weighted feature map: $F_{k}=A_{k}⊙F$10:                  Apply global average pooling on $F_{k}$ to obtain feature vector $f_{k}$11:            **end for**12:            Concatenate features: $F_{m}=\left[f_{1};f_{2};\ldots ;f_{M}\right]$13:            Compute raw prediction: $p_{\mathrm{raw}}=\mathrm{linear}\left(F_{m}\right)$14:            Update feature center: $C\left[y\right]=\left(1-\beta \right)C\left[y\right]+\beta F_{m}$15:            Randomly select two attention maps $A_{1},A_{2}$ from *A*16:            Apply attention cropping/dropping on $A_{1},A_{2}$ to obtain $X_{\mathrm{crop}},X_{\mathrm{drop}}$17:            Feed into model to obtain $p_{\mathrm{crop}},p_{\mathrm{drop}}$18:            Compute average cross-entropy loss:$$L_{a}=\frac{1}{3}\left(L_{ce}\left(p_{\mathrm{raw}},y\right)+L_{ce}\left(p_{\mathrm{drop}},y\right)+L_{ce}\left(p_{\mathrm{crop}},y\right)\right)$$19:            Compute feature center loss:$$L_{c}=\mathrm{MSELoss}\left(F_{m},C\left[y\right]\right)$$20:            Compute total loss: $L_{\mathrm{total}}=L_{a}+\lambda L_{c}$21:            Backpropagate $L_{\mathrm{total}}$22:            Update parameters θ using SGD23:            Adjust learning rate α using StepLR scheduler24:      **end for**25:**end for**

To derive a unified video-level classification from frame-level fake probabilities $p_{n}\in \left[0,\;1\right]$, where n=1,…,N corresponds to sampled frames, we propose an aggregation strategy that balances sensitivity to forgery cues with robustness against noise. A straightforward yet effective approach is to first count the number of frames where $p_{n}$ exceeds a predetermined detection threshold τ. If any such frame exists, the video is immediately classified as fake according to the rule

(6) $$D=\left\{\begin{array}{cc} 1, & \max_{n}p_{n}\geq \tau ,\\ 0, & \max_{n}p_{n}<\tau , \end{array}\right.$$

thereby maximizing sensitivity to even a single high-confidence forgery cue. However, this “any-frame” criterion can be overly susceptible to occasional false alarms, so we complement it with an average-confidence criterion that computes the mean probability $\bar{p}=\frac{1}{N}\sum_{n=1}^{N}p_{n}$ and labels the video as fake only if $\bar{p}$ exceeds a global threshold γ,

(7) $$D=\left\{\begin{array}{cc} 1, & \bar{p}\geq \gamma ,\\ 0, & \bar{p}<\gamma . \end{array}\right.$$

This averaging strategy dilutes sporadic outliers and is particularly effective when forgeries manifest across multiple frames.

To further mitigate computational load in long sequences, we restrict evaluation to a smaller subset of “key frames” ${\left\{n_{k}\right\}}_{k=1}^{K}$, selected for their high motion or salience. In our implementation, key frames are identified based on frame-level prediction confidence, where frames with the highest forgery scores $p_{n}$ are selected. We empirically set K=8 as a balance between detection robustness and computational efficiency. A soft majority vote is then performed by requiring the proportion of key frames with $p_{n_{k}}\geq \tau$ to exceed a ratio ρ:

(8) $$D=\left\{\begin{array}{cc} 1, & \frac{1}{K}\sum_{k=1}^{K}1\left\{p_{n_{k}}\geq \tau \right\}\geq \rho ,\\ 0, & \mathrm{otherwise}, \end{array}\right.$$

where 1{·} denotes the indicator function.

Beyond these rules, more flexible schemes are incorporated. In particular, weighted aggregation assigns higher importance to frames with stronger attention responses or higher face detection confidence, while a hierarchical strategy first flags suspicious frames using the max rule and then confirms them through averaging or voting.

By integrating these complementary aggregation rules—max-based detection, mean confidence, key-frame voting, and weighted extensions—into a cohesive decision framework, our system forms a natural continuation of the attention-guided frame-level design, ensuring that localized forgery cues are effectively elevated to a reliable and stable video-level verdict.

The thresholds are not chosen from the test set. In our implementation, τ, γ, *K*, and ρ are selected on the validation split by grid search with two objectives: maximizing validation AUC/ACC, and avoiding excessive false positives on real videos. The final values are then fixed for all test datasets. This calibration protocol reduces the risk that the reported performance is tied to a single favorable threshold, and it supports a fairer comparison between the sensitivity-oriented and stability-oriented decision rules.

### 3.3. Preprocessing

To guarantee that the model receives clean, focused, and geometrically standardized inputs, we designed a preprocessing pipeline that transforms raw videos into aligned facial images, which are suitable for precise deepfake detection. This step is crucial because most manipulations in deepfake videos occur within facial regions. Proper localization and normalization enable the network to concentrate on areas of semantic significance, ensuring more accurate and effective results.

The preprocessing process begins with uniform frame sampling. For each video, this method extracts one frame per second—a frequency selected to strike a balance between temporal diversity and computational efficiency, which guarantees that a sufficient number of frames are sampled to reveal potential forgeries while minimizing unnecessary redundancy among highly similar adjacent frames.

Next, each sampled frame is processed through the RetinaFace detector. RetinaFace is a single-stage dense facial detection network, particularly suitable for our task due to its robustness in handling extreme head poses, occlusion, and low resolution. For each frame, this method retains the largest detected facial bounding box, assuming that the subject of interest typically occupies the most prominent area of the frame. To ensure that contextual information, such as the cheeks and jawline, is not truncated, the bounding box is expanded by a fixed scale factor of 1.3 along both axes, which enlarges the region and then serves as the candidate face region.

For implementation consistency, face localization is performed through the InsightFace face analysis interface with a RetinaFace-style detector. When multiple faces are detected, the largest face is selected because the benchmark videos are dominated by a principal subject. When no face is detected in a sampled frame, the frame is skipped and the decision is made from the remaining valid frames; if no valid face frame is obtained for a video, the video is excluded from quantitative scoring and recorded as a preprocessing failure. This policy prevents low-quality frames, missed detections, or background faces from silently biasing the model prediction.

To mitigate the variation introduced by different face orientations and camera angles, this method applies facial alignment using five key landmarks provided by RetinaFace: the centers of the left and right eyes, the nose tip, and the corners of the mouth. Let the detected landmarks in the input image be denoted as $P=\{p_{1},p_{2},p_{3},p_{4},p_{5}\}$, and let the reference template landmarks be $Q=\{q_{1},q_{2},q_{3},q_{4},q_{5}\}$. We then compute a similarity transformation *T* that minimizes the least-squares alignment error:

(9) $$T^{*}=\arg\min_{T}\sum_{i=1}^{5}{∥T\left(p_{i}\right)-q_{i}∥}^{2}.$$

This transformation is applied to warp the detected face region so that the five landmarks align with a canonical configuration, resulting in consistent geometric alignment across all samples.

After alignment, each facial image is cropped and resized to a fixed spatial resolution of 300×300 pixels to meet the input specifications of the backbone architecture. Subsequently, the pixel intensities are rescaled to the range of [0, 1], followed by channel-wise normalization utilizing the mean and standard deviation values derived from the ImageNet dataset:

(10) $$I_{\mathrm{norm}}\left(x,y\right)=\frac{I(x,y)-\mu }{\sigma },\;\mu ,\sigma \in R^{3},$$

where μ and σ are the per-channel mean and standard deviation vectors used during EfficientNet pretraining.

Consequently, each input video is transformed into a sequence of geometrically aligned, photometrically normalized facial images that maintain consistent resolution and structured semantics. These processed face crops are then fed into the network for feature extraction and classification, forming the foundation for dependable and robust deepfake detection.

### 3.4. Data Augmentation

To enhance the robustness of the model and improve its ability to focus on discriminative local features, we propose an attention-guided data augmentation strategy inspired by the WAFF framework. Unlike traditional data augmentation techniques that apply global transformations such as random flipping, color jittering, or cropping, our approach leverages spatial attention maps derived from the model to dynamically generate augmentation masks. These attention maps highlight the most informative regions of an input image, allowing for the targeted amplification or suppression of local features during training.

Formally, given a feature map $F\in R^{B\times C\times H\times W}$ extracted from the backbone and its corresponding attention maps $A\in R^{B\times M\times H\times W}$, two attention maps are selected for each training image: one for cropping and one for dropping. Let $A_{k}\in R^{H\times W}$ denote the *k*-th attention map from the set. For cropping, we identify the bounding region where the attention intensity exceeds a threshold $\theta_{c}$ and extract the corresponding image patch:

(11) $$\mathrm{CropRegion}\left(A_{k}\right)=\left\{\left(i,j\right)|A_{k}\left(i,j\right)>\theta_{c}\right\}$$

Then, the corresponding image patch is resized to the original image resolution. This approach necessitates that the model prioritizes the examination of prominent local features, including the eyes, mouth, and facial contours, and concentrates on these salient areas that are often most vulnerable to the introduction of forgery artifacts.

Conversely, for attention dropping, we mask out a high-attention region by setting the corresponding pixels to zero or a neutral value, effectively encouraging the network to explore alternative regions:

(12) $$I^{\prime }=I\cdot \left(1-I_{A_{k}>\theta_{d}}\right),$$

where I denotes the binary mask induced by a drop threshold $\theta_{d}$, and *I* is the original input image. This operation aims to prevent the model from overfitting to only the most salient patterns and promotes the learning of complementary features.

To balance these two augmentation strategies, we randomly sample two attention maps $A_{i}$, $A_{j}$ from the top-activated set for each mini-batch sample. One produces a cropped augmented image, and the other generates a dropped version. Both are included in the training batch with the original image, which increases intra-class variance and creates a more diverse and challenging training distribution.

## 4. Experiments and Discussion

### 4.1. Experimental Setup

#### 4.1.1. Dataset and Preprocessing

In this section, for the sake of convenient verification and fair comparison, we adhere to the experimental settings described in the previous IJCV paper [18]. Specifically, we adopt FaceForensics++ (FF++) as the primary training dataset. FaceForensics++ originally consists of 1000 pristine source videos collected from YouTube, which have subsequently been manipulated via four widely recognized techniques: DeepFakes (DF), FaceSwap (FS), Face2Face (F2F), and NeuralTextures (NT). In its extended release, an additional state-of-the-art face-swapping method, named FaceShifter, has also been incorporated to further increase the diversity and realism of the forged samples. This new manipulation type is likewise included in our experimental setup and evaluation. The above manipulated videos were sourced from multiple forgery techniques to showcase a wide array of artifacts, guaranteeing that the dataset reflects a diverse range of manipulation methods, such as identity swaps, expression reenactments, and advanced deepfake techniques, while also ensuring diversity in actors, lighting conditions, and backgrounds. Moreover, each video in FF++ is available under different compression levels: RAW (lossless), c23 (high quality), and c40 (low quality). In line with the IJCV setup, and to maintain consistency with prior studies, we adopted the c23 compressed version of the dataset for training unless otherwise specified. Furthermore, following common practice, the dataset was divided into training, validation, and testing subsets at a ratio of 7:1:2, ensuring a balanced and standardized evaluation protocol.

After splitting, each video in the training and validation sets underwent a standardized preprocessing pipeline. First, OpenCV was used to uniformly sample N=10 frames per video at one-frame-per-second intervals, with each frame saved as an individual image and initially resized to ensure its width fell within a range suitable for face detection. Next, a pretrained RetinaFace detector with a ResNet-50 backbone was applied to locate facial bounding boxes, from which the largest face was selected to enhance robustness. To retain contextual features such as the cheeks and forehead, the selected bounding box was further expanded by a factor of 1.3 around its center. Finally, the expanded region was cropped from the original frame, resized to 300×300 pixels for consistency, and stored in the corresponding directories for each dataset split (training, validation, and testing) and class label (real or fake).

For evaluation, we further conducted cross-dataset testing on several widely used benchmarks, including Celeb-DF v2 (CDF), Deepfake Detection (DFD), DFDC and its preview version (DFDC-P), and FFIW-10K. The characteristics and real/fake distributions of these datasets are summarized in Table 2.

#### 4.1.2. Training Details

The training was conducted on a server equipped with an NVIDIA A40 GPU and an Intel(R) Xeon(R) Gold 6346 CPU running at 3.10 GHz with 16 cores and 32 threads, on a 64-bit Linux system within a KVM virtualized environment. The software stack included Python 3.10.12 and PyTorch 2.12 compiled with CUDA 12.6 support.

All trainable parameters, including the EfficientNet-B3 backbone and WS-DAN attention modules, were optimized using stochastic gradient descent (SGD) with momentum. A summary of the training hyperparameters and WS-DAN augmentation settings is provided in Table 3. The training process spanned 20 epochs with a batch size of 16, employing a composite loss function combining cross-entropy and center loss with a center loss weight of 0.05. A cosine annealing learning rate scheduler was used to adjust the learning rate dynamically, starting from an initial value of 0.001. For WS-DAN, 4 attention maps were generated for each image, with attention cropping applied at thresholds between 0.3 and 0.7, and attention dropping applied at thresholds between 0.3 and 0.6. Early stopping was implemented with a patience of 7 epochs based on validation accuracy.

**Figure 2:**
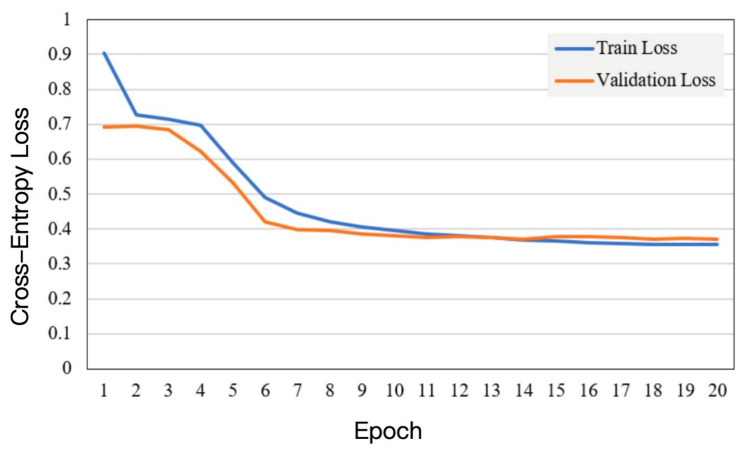
Training cross-entropy loss curve over epochs.

**Figure 3:**
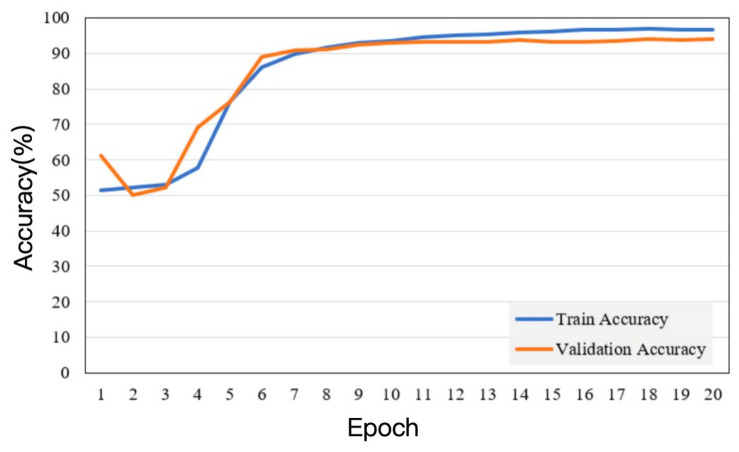
Training accuracy curve over epochs.

As illustrated in Figure 2 and Figure 3, the training process exhibits stable convergence. The cross-entropy loss decreases steadily during the first 10 epochs, followed by a plateau as the learning rate decays under the cosine annealing schedule. This behavior indicates that the model effectively minimizes the objective without oscillations or divergence. The training accuracy curve follows a complementary trend, rising sharply in the early epochs and gradually approaching saturation. Notably, the accuracy stabilizes around epoch 15, which aligns with the early stopping criterion and prevents overfitting while preserving detection performance. These observations confirm that the chosen hyperparameter configuration and optimization strategy strike a good balance between convergence speed and stability, ensuring reliable training of the WSEffiNet network with WS-DAN attention.

#### 4.1.3. Evaluation Metrics

Following the widely adopted evaluation protocol in the field, we assessed the performance of our proposed method using two widely adopted metrics: accuracy (ACC), and area under the receiver operating characteristic curve (AUC). These complementary measures collectively capture both the correctness of pointwise predictions at a chosen decision threshold and the classifier’s discrimination capability across all possible thresholds, thereby providing a balanced view of detection quality including correctness, separability, and the trade-off between false positives and false negatives.

Accuracy (ACC): Accuracy quantifies the proportion of correct predictions made by the classifier at a specific decision threshold. In the binary forgery detection setting (real vs. fake), ACC is calculated as follows:

(13) $$\mathrm{ACC}=\frac{TP+TN}{TP+TN+FP+FN},$$

where TP (true positives) is the number of forged samples correctly identified as forged, TN (true negatives) is the number of pristine samples correctly identified as real, FP (false positives) is the number of pristine samples incorrectly flagged as forged, and FN (false negatives) is the number of forged samples missed by the detector. ACC is simple and intuitive: a higher ACC indicates a larger fraction of correct decisions under the chosen threshold. However, it depends on the selected decision threshold (commonly 0.5 when using probabilistic outputs) and can be misleading in the presence of severe class imbalance—in such cases, a high ACC might be achieved by a classifier that favors the majority class.

Area Under the Curve (AUC): AUC evaluates the classifier’s ability to rank positive (forged) instances higher than negative (real) ones over the full range of classification thresholds. It is defined as the area under the receiver operating characteristic (ROC) curve, where the ROC curve plots the True Positive Rate (TPR) against the False Positive Rate (FPR) as the decision threshold varies:

(14) $$\mathrm{TPR}=\frac{TP}{TP+FN},\;\mathrm{FPR}=\frac{FP}{FP+TN}.$$

The AUC score ranges from 0 to 1. An AUC of 0.5 indicates no discriminative ability (equivalent to random guessing), while an AUC of 1.0 corresponds to a perfect ranking (all forged samples ranked above all real samples). Because AUC aggregates performance across all thresholds, it is threshold-independent and generally more robust to class imbalance than ACC. Intuitively, AUC can also be interpreted as the probability that the classifier assigns a higher score to a randomly chosen forged sample than to a randomly chosen real sample.

In practice, we report both ACC and AUC for in-dataset evaluations, as they provide complementary information: ACC reflects actual decision performance at a selected operating point (useful for deployment and real-time systems), while AUC reflects the intrinsic separability and ranking quality of the learned scoring function (useful for comparing models independent of a specific threshold). For cross-dataset evaluations, however, we focus exclusively on AUC. This is because cross-dataset settings typically involve substantial distribution shifts and unseen manipulation techniques, where ACC under a fixed threshold may not be meaningful, while AUC provides a more reliable and threshold-independent measure of generalization ability.

In short, higher ACC and AUC values jointly indicate stronger forgery detection performance: ACC shows pointwise correctness under a given threshold in in-dataset settings, while AUC demonstrates robust discrimination ability across thresholds, especially in cross-dataset evaluations where threshold-free comparison is essential.

### 4.2. Performance Evaluation

**Figure 4:**
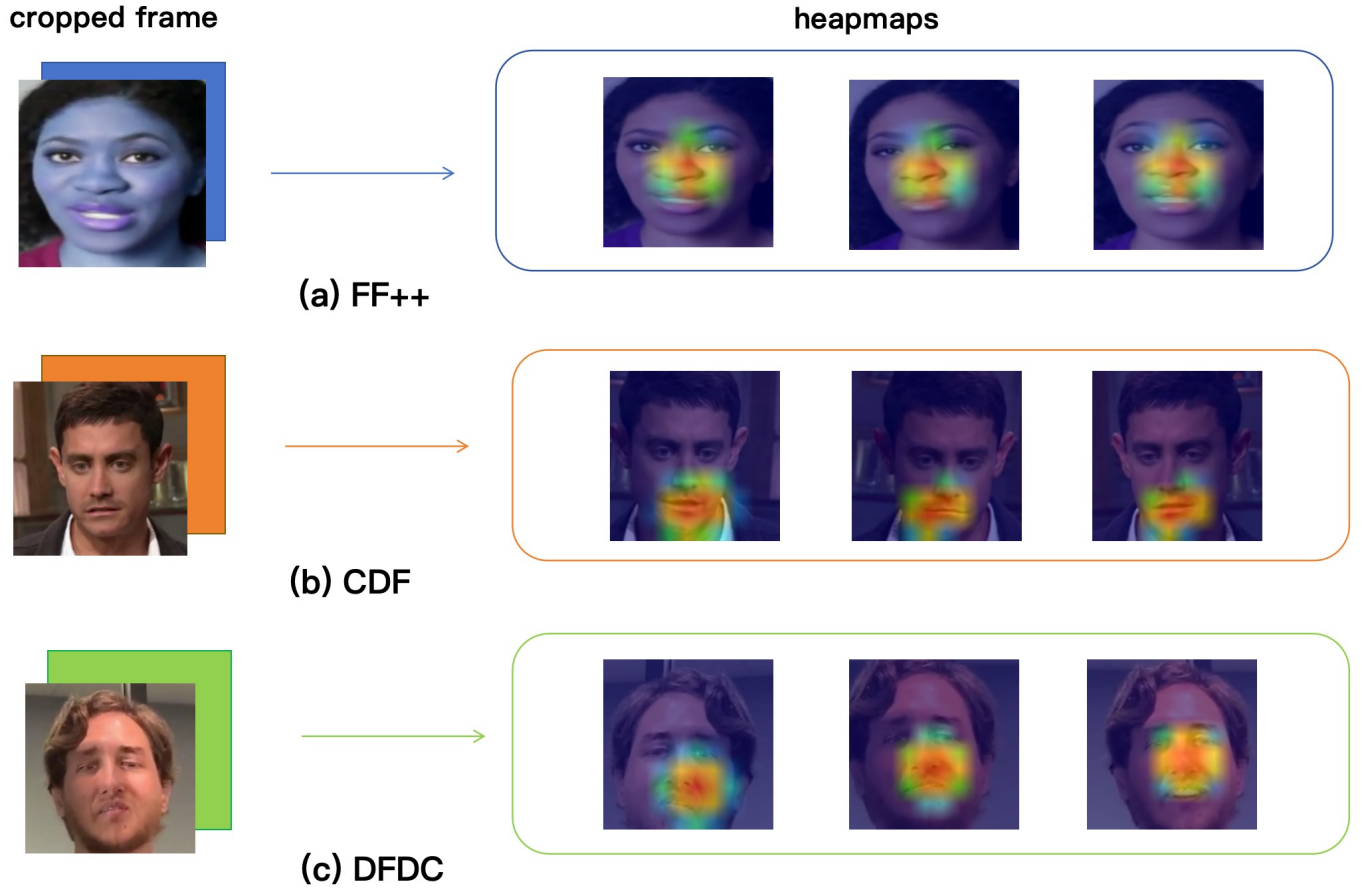
Heatmap visualizations of WAFF across three datasets: (**a**) FF++, illustrating the model’s localized responses to manipulated facial regions; (**b**) CDF, showing robust cross-dataset localization under domain shift; and (**c**) DFDC, further demonstrating cross-dataset robustness in the presence of diverse visual conditions and compression levels.

As shown in Figure 4, the proposed prediction model WSEffiNet is able to capture discriminative artifacts by focusing on key facial regions, particularly around the nose and mouth. Across the most representative datasets, including FF++, CDF, and DFDC, the heatmaps consistently highlight these areas as the most informative for distinguishing real from forged content. This indicates that WSEffiNet not only achieves accurate detection but also provides interpretable attention patterns aligned with known forgery cues, thereby validating the robustness and generalization ability of the model.

These highlighted regions correspond to facial areas that are particularly vulnerable to deepfake generation processes. In practice, blending operations and identity transfer often introduce subtle inconsistencies around facial boundaries and key components such as the mouth and nose, where precise alignment and texture synthesis are challenging. The attention responses therefore offer an intuitive explanation of how WSEffiNet detects forged frames by emphasizing localized visual anomalies, rather than relying solely on global appearance statistics.

#### 4.2.1. In-Dataset Evaluation

As presented in Table 4, our proposed method is evaluated on the FaceForensics++ dataset under both high-quality (HQ, c23) and low-quality (LQ, c40) compression settings. In the HQ scenario, our approach attains the highest classification accuracy, at 98.87%, outperforming all compared baselines, while achieving the second-best AUC at 98.42%, slightly below FD-GAN (99.75%). This indicates that our proposed method exhibits superior performance in terms of precise classification at fixed thresholds, whereas FD-GAN demonstrates a marginal advantage in threshold-independent ranking capability. In the more challenging LQ setting, our method achieves the best accuracy (93.21%), considerably surpassing the second-best competitor FD-GAN (91.60%), which highlights its robustness under degraded visual quality. Although the AUC of our model (96.57%) is slightly lower than that of FD-GAN and Two Branch, it remains highly competitive and demonstrates strong discriminative ability. Overall, these results suggest that the proposed method consistently achieves state-of-the-art performance in terms of accuracy across different compression levels, while maintaining competitive AUC values, thereby confirming its effectiveness and robustness for in-dataset forgery detection.

**Figure 5:**
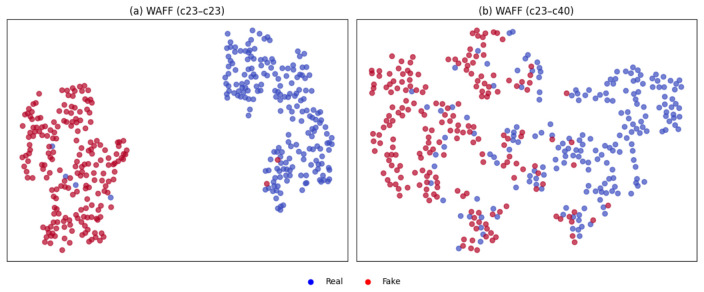
The t-SNE visualizations of feature embeddings extracted by WAFF: (**a**) features from FF++ (c23→c23), showing clear separation between real and fake samples; and (**b**) features under cross-compression (c23→c40), illustrating that the separation remains stable despite increased compression and distribution shift.

Beyond quantitative metrics, we further provide qualitative insights through t-SNE visualizations of the learned feature representations, as shown in Figure 5. In the c23–c23 setting, where the model is trained and tested on videos compressed at c23, real and fake instances concentrate in two distinct regions of the embedding space; nevertheless, a small subset of samples are not merely boundary cases but are embedded within the opposing class cluster, which accounts for the few definitive misclassifications observed numerically. This demonstrates that our model learns highly discriminative embeddings under high-quality conditions. In contrast, in the c23–c40 setting, where the model is trained on c23 and tested on c40, real and fake samples remain largely distinguishable, although their distributions exhibit a partial overlap, reflecting the inherent challenges posed by stronger compression. Nonetheless, even in the presence of such overlap, the two classes still maintain distinct regions in the embedding space, indicating that the proposed model effectively captures discriminative cues despite quality degradation. These visual results further corroborate the quantitative findings, confirming both the robustness and the generalization ability of our method across different compression scenarios.

#### 4.2.2. Cross-Dataset Evaluation

As shown in Table 5, our proposed WAFF framework consistently achieves superiority across multiple cross-dataset evaluations, highlighting its robustness and generalization capabilities. Specifically, WAFF achieves the best performance on all five benchmark datasets, with results of 87.43% on CDF, 96.02% on DFD, 79.63% on DFDC, 82.57% on DFDC-P, and 74.30% on FFIW. Compared with classical frame-level detectors such as Xception and Face X-ray, the proposed WAFF framework exhibits clear margins of 5–10% on average, demonstrating its ability to adapt to unseen forgery techniques and diverse data distributions.

It is also noteworthy that frame-based methods like CADDM and UIA-ViT perform competitively on single datasets, but their performance drops substantially in more challenging scenarios such as DFDC and FFIW. This performance degradation can be attributed to the limited capacity of frame-only modeling, which often overfits dataset-specific artifacts and fails under unseen compression patterns or manipulation pipelines. In contrast, WAFF maintains balanced improvements across all datasets. The primary reasons for this are twofold: first, the attention-guided augmentation strategy forces the network to learn complementary local–global features, mitigating over-reliance on dataset-specific cues; second, the multi-strategy video-level fusion robustly aggregates frame-level predictions, reducing the influence of spurious detections and enhancing stability across heterogeneous datasets.

Overall, these results demonstrate that WAFF not only surpasses state-of-the-art baselines under in-distribution conditions but also generalizes well to out-of-distribution datasets. The consistent performance gains validate that the proposed framework effectively addresses key challenges such as cross-dataset generalization and robustness against compression, noise, and unseen manipulations, thereby making it highly suitable for real-world forensic applications.

#### 4.2.3. Generation-Type and Error-Case Diagnostics

In addition to the overall results, we further separate the FF++ evaluation according to the underlying manipulation type, including DeepFakes, FaceSwap, Face2Face, NeuralTextures, and FaceShifter. This generation-type view is important because different forgery algorithms leave different traces: identity-swap methods often introduce boundary and texture-blending artifacts, whereas expression-reenactment methods may produce weaker spatial artifacts but stronger motion or mouth-region inconsistencies. Reporting results in this form helps distinguish whether the detector is only effective on legacy manipulation pipelines or remains useful for newer and more realistic generation methods.

To provide an additional lightweight diagnostic under local computational constraints, we conducted a small-scale FF++ subset test using RGB frames from five manipulation methods. This experiment was intended to complement the main benchmark results by showing relative behavior across generation types; it was not used as the primary state-of-the-art comparison. As shown in Table 6, the detector exhibits different sensitivity across manipulation families, with DeepFakes achieving the highest subset AUC, while NeuralTextures is more challenging in this lightweight setting.

We also inspected false positive and false negative cases at the video level. False positives are mainly associated with low-resolution real videos, strong compression, motion blur, and extreme head pose, where natural facial texture degradation resembles forgery artifacts. False negatives are more likely to occur when manipulated videos are heavily compressed, when only a few sampled frames contain visible artifacts, or when the forged region is small and temporally intermittent. These observations support the need for the proposed fusion design: max-based rules improve sensitivity to sparse forged frames, whereas confidence averaging and attention-guided voting reduce false alarms caused by isolated unreliable frames.

Table 7 further summarizes how different video-level fusion rules affect FP/FN behavior in the same lightweight diagnostic setting. The key-frame voting rule produces the highest subset accuracy and the lowest false negative rate, indicating stronger sensitivity to sparse manipulated frames. Confidence averaging is more conservative, reducing false positives but missing more fake videos. This pattern is consistent with the design motivation that different aggregation rules trade sensitivity against stability.

To make the diagnostic evaluation reproducible, the accompanying source code records per-video frame probabilities, valid-frame counts, prediction statistics, and FP/FN labels. These logs can be used to generate manipulation-specific tables, threshold-sensitivity curves, and representative heatmap visualizations for failure analysis.

#### 4.2.4. Robustness and Deployment Considerations

Beyond the c23 and c40 compression settings, practical deployment may involve resizing, recompression, blur, sensor noise, and missing or multiple faces. WAFF addresses part of this variability through face alignment, ImageNet normalization, attention-guided augmentation, and validation-calibrated video fusion. Nevertheless, severe perturbations can still reduce the reliability of frame-level probabilities. Therefore, we regard the video-level fusion module as a deployment-oriented stabilizer rather than a replacement for robust preprocessing.

For real-time use, the main computational cost comes from face detection, repeated frame inference, and attention-guided refinement. Increasing the number of sampled frames improves temporal coverage but yields diminishing returns because adjacent frames are often redundant. In practice, the sampling rate should be selected according to the latency budget: fewer frames are preferable for online screening, whereas denser sampling is appropriate for offline forensic analysis.

### 4.3. Ablation Study

#### 4.3.1. Backbone Model Selection

The backbone network serves as the feature extractor in deepfake face video detection models and largely determines detection accuracy, runtime efficiency, and generalization capability. Selecting an appropriate backbone is therefore crucial to balance performance and computational cost in practical scenarios.

To evaluate candidate backbones, we compared representative models with respect to three indicators: classification accuracy, parameter count, and FLOPs. For accuracy assessment, we constructed a balanced dataset by sampling 500 real and 500 fake videos from FF++, CDF, and DFDC-P, together with image-level samples from the DFW dataset. All models were trained and evaluated under the same preprocessing and training pipeline to ensure a fair comparison.

The results presented in Table 8 show that EfficientNet models achieve competitive or superior accuracy with substantially fewer parameters and FLOPs compared to deeper backbones such as ResNet-152, Inception-v4, or SENet. In particular, EfficientNet-B3 provides an optimal trade-off, reaching 81.1% accuracy with only 12M parameters and 1.8B FLOPs. This demonstrates that, despite its moderate depth, EfficientNet-B3 is capable of capturing subtle forgery artifacts such as boundary inconsistencies and texture anomalies, while maintaining efficient inference suitable for real-time detection. Considering these results and the constraints of available computational resources, we adopted EfficientNet-B3 as the backbone for the proposed WAFF framework, rather than larger but more resource-intensive alternatives.

#### 4.3.2. Impact of Decision Rules

We evaluated four video-level aggregation strategies on a mixed benchmark constructed by sampling 500 real and 500 fake videos from each of FF++ (c23), Celeb-DF, and DFDC-P (total: 1500 real + 1500 fake). The compared rules were fake-frame counting (FFC), confidence averaging (CA), key-frame voting (KFV), and our proposed Attention-Guided Fusion Decision (AGFD) introduced in Section 3.2. For fairness, the same trained WAFF frame-level model was used for all methods; per-frame threshold τ=0.6, averaging threshold γ=0.5, and KFV selects K=8 key frames with majority ratio ρ=0.5.

The results in Table 9 reveal a clear hierarchy among the four decision rules. FFC performs worst, as its “any-frame” criterion makes it overly sensitive to isolated high-confidence predictions, which boosts recall for sporadic manipulations but also amplifies false positives, leading to the lowest AUC. CA mitigates this issue by averaging frame scores, producing smoother and more stable decisions that improve both ACC and AUC over FFC, although it can weaken detection when forgeries appear only in a few frames. KFV further refines performance by restricting aggregation to salient frames, thereby retaining sensitivity to informative cues while filtering out irrelevant noise, resulting in modest gains over CA. Our proposed AGFD achieves the most substantial improvement, with a clear margin in both ACC and AUC. By weighting frames according to attention maps and combining soft aggregation with a calibrated voting mechanism, AGFD emphasizes spatially coherent forgery cues while suppressing background noise and transient artifacts, striking an effective balance between sensitivity and robustness. This design not only enhances the detection of subtle manipulations but also reduces false alarms, explaining the consistent superiority of AGFD over conventional rules and justifying its adoption as the default aggregation strategy in WAFF.

#### 4.3.3. Effect of Attention-Guided Augmentation

**Figure 6:**
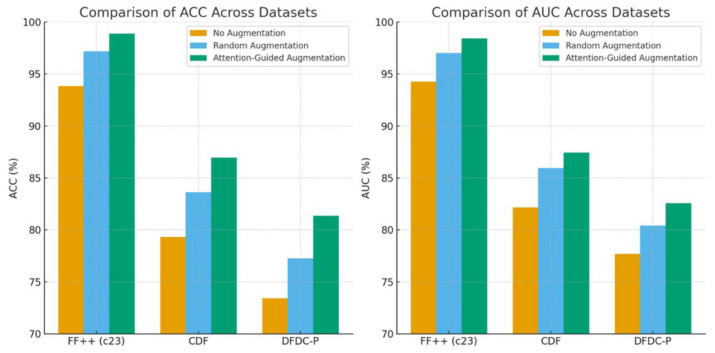
Ablation study on augmentation strategies across different datasets.

To evaluate the influence of augmentation strategies on both in-dataset performance and cross-dataset generalization, we conducted controlled ablation experiments on three benchmarks: FF++ (c23), Celeb-DF (CDF), and DFDC-P. For each benchmark, we sampled 500 real and 500 fake videos and trained the models under the same preprocessing and optimization pipeline. We compared three augmentation regimes: (1) no augmentation beyond standard preprocessing, (2) conventional random augmentation (random crop, horizontal flip, color jitter), and (3) attention-guided augmentation (attention-driven cropping and dropping, as described in Section 3.4). Classification accuracy (ACC) and the area under the ROC curve (AUC) are reported for each dataset, as illustrated in Figure 6.

The results in Figure 6 clearly demonstrate that augmentation strategies substantially influence detection performance. Without augmentation, the model exhibits noticeable performance degradation, especially under cross-dataset evaluation, where the ACC drops below 80% on CDF and 75% on DFDC-P. This indicates that models trained without augmentation tend to overfit dataset-specific artifacts and fail to generalize to unseen manipulations. Incorporating random augmentation mitigates this problem by introducing data diversity, leading to moderate improvements of around 4–5% in both ACC and AUC.

In contrast, attention-guided augmentation consistently achieves the best results across all benchmarks. On FF++ (c23), its performance is further refined to 98.87% ACC and 98.42% AUC. More importantly, substantial gains are observed in cross-dataset evaluations, where the ACC increases to 86.94% on CDF and 81.35% on DFDC-P, with the AUC reaching 87.43 and 82.57, respectively. These improvements highlight the effectiveness of leveraging attention maps to guide cropping and dropping operations, which forces the model to focus on subtle but transferable forgery cues such as boundary artifacts and unnatural micro-expressions, while reducing reliance on background or dataset-specific textures.

Overall, the attention-guided strategy not only ensures state-of-the-art accuracy on in-dataset evaluations but also significantly enhances robustness in cross-dataset settings, validating its role as an essential component of the WAFF framework.

#### 4.3.4. Weak vs. Full Supervision

To examine the role of weak supervision in the proposed WAFF framework, we introduced a fully supervised variant, denoted as WAFF-FS. Following the mask-based supervision paradigm commonly adopted in previous works, pixel-level pseudo-artifact masks were generated using the Face X-ray pipeline, and a region-consistency loss was added to incorporate these masks into training. This formed a conventional fully supervised setting in which explicitly defined spatial supervision constrains the feature learning process. In contrast, the weakly supervised configuration of WAFF relies on WS-DAN attention, which produces spatial responses from image-level labels without using region annotations or pixel-level artifact masks.

Table 10 summarizes the comparison between weak and full supervision across three benchmark datasets.

The fully supervised variant achieves a slight performance increase on FF++ (+0.42 AUC), indicating that explicit spatial supervision improves in-dataset fitting. However, it shows noticeably reduced generalization, with performance drops of 3.31 AUC on Celeb-DF and 5.73 AUC on DFDC-P. This effect aligns with prior observations that mask-based supervision tends to encode dataset-specific manipulation characteristics, which may hinder transferability to unseen or heterogeneous forgery distributions.

In contrast, the weakly supervised design of WAFF relies on spatial cues implicitly derived from WS-DAN attention, allowing the model to focus on informative regions while maintaining flexibility in learning transferable representations. The results indicate that weak supervision provides a more favorable balance between dataset-level fitting and cross-dataset robustness.

#### 4.3.5. Frame Sampling and Preprocessing Strategies

Frame sampling and preprocessing not only affect detection accuracy but also influence efficiency and prediction stability. To capture these aspects more comprehensively, we evaluated two complementary indicators: inference time (IT) and prediction variance (PV). IT measures computational efficiency as the average processing time per video (including face extraction and model inference). PV quantifies intra-video prediction stability by computing the variance of frame-level confidence scores within each video; a lower PV indicates more consistent decisions across frames.

From Table 11, we can observe several important trends regarding the influence of frame sampling and preprocessing. First, increasing the sampling rate from 1 fps to 2 fps and 4 fps only marginally reduces the PV (from 0.021 to 0.019), indicating slightly more stable predictions across frames. However, this comes at the cost of a dramatic increase in inference time, rising from 120 ms to 380 ms per video. These results suggest that higher frame rates introduce a large amount of redundant information, since consecutive frames often contain highly similar content and forgery artifacts. As a result, the additional computational burden does not translate into meaningful stability improvements. Therefore, 1 fps emerges as the most efficient choice, already providing sufficient temporal coverage while keeping the computational cost low, which is particularly valuable in real-time or resource-constrained deployment scenarios.

Moreover, the removal of geometric alignment has a much more severe impact. While the inference time decreases slightly to 95 ms per video, the PV more than doubles from 0.021 to 0.047, reflecting highly unstable predictions within the same video. This instability can be attributed to uncontrolled variations in face scale, position, and pose across frames, which cause the detector to focus on inconsistent spatial regions rather than forgery-specific cues. Consequently, alignment is indispensable for normalizing facial geometry and stabilizing the spatial correspondence of features, thereby ensuring robust frame-level consistency and reducing the risk of overfitting to dataset-specific artifacts.

## 5. Conclusions

This paper introduces WAFF, a novel deepfake detection framework that integrates WSEffiNet with a flexible video-level fusion strategy. WAFF leverages an EfficientNet-B3 backbone enhanced by a Weakly Supervised Data Augmentation Network (WS-DAN) to generate fine-grained attention maps, which highlight subtle forgery artifacts. At the video level, we further propose an attention-guided decision fusion mechanism that effectively balances sensitivity to localized manipulations with robustness against spurious noise and compression artifacts. Extensive experiments across multiple benchmarks, including FaceForensics++, Celeb-DF, DFDC, DFD, and FFIW-10K, demonstrate that WAFF consistently achieves superior in-dataset performance and outperforms state-of-the-art methods in challenging cross-dataset evaluations. These results confirm the robustness, generalization ability, and efficiency of WAFF for practical deployment in real-world deepfake forensics.

The current framework also has limitations. First, WAFF depends on reliable face detection and alignment; severe occlusion, very small faces, or unusual camera views may reduce the number of valid frames and weaken video-level confidence. Second, although EfficientNet-B3 offers a favorable accuracy–efficiency trade-off, the model still benefits from ImageNet pretraining and may require additional adaptation when deployed against substantially different attack families. Third, video-level aggregation adds latency compared with a single-frame classifier, especially when dense sampling is used. Finally, the present design primarily models frame-level evidence with lightweight aggregation rather than explicit long-range temporal dynamics, which may limit sensitivity to manipulations that are visible only through extended motion inconsistencies.

In future work, we aim to explore several promising directions to further advance this line of research. First, we plan to investigate more lightweight architectures and model compression techniques to reduce inference costs and enable deployment on diverse edge devices. Second, we will incorporate adaptive temporal modeling strategies to better capture long-range dynamics in videos without sacrificing efficiency. Finally, we also plan to study explainable deepfake detection by developing visualization tools that explicitly reveal discriminative artifacts, which may enhance interpretability and trustworthiness in forensic practice.

## Figures and Tables

**Table 1:** Architecture of WSEffiNet.

Stage	Module	Input Size	Output Channels	Layers
1	Conv 3×3	300×300	40	1
2	MBConv1 (k=3)	150×150	24	3
3	MBConv6 (k=3)	150×150	32	3
4	MBConv6 (k=5)	75×75	48	3
5	MBConv6 (k=3)	38×38	96	5
6	MBConv6 (k=5)	38×38	136	5
7	MBConv6 (k=5)	19×19	232	6
8	MBConv6 (k=3)	10×10	384	1
9	Conv 1×1	10×10	1536	1
10	WSDAN Attention	10×10	—	—
11	Global Pooling → FC	10×10	$num_{classes}$	1

**Table 2:** Summary of external datasets used for evaluation.

Dataset	Manipulation Techniques	Real/Fake (Videos)
CDF-v2	High-quality face swapping	590/5049
DFD	Google-created face swaps	363/3005
DFDC	Mixed methods (FaceSwap, GANs, etc.)	23,654/104,500
DFDC-P	Early subset of DFDC	1131/4113
FFIW-10K	Wild deepfakes with compression	5000/5000

**Table 3:** Training hyperparameters and settings for the WSEffiNet model.

Module	Parameter/Item	Value
Training	Batch size	16
	Number of epochs	20
	Loss function	Cross-entropy
	Center loss weight β	0.05
	Optimizer	SGD
	Initial learning rate	0.001
	Momentum	0.9
	Weight decay	5 × $10^{-4}$
	Learning rate scheduler	CosineAnnealingLR
WS-DAN	Number of attention maps *M*	4
	Cropping threshold $\theta_{c}$	(0.3, 0.7)
	Dropping threshold $\theta_{d}$	(0.3, 0.6)

**Table 4:** In-dataset evaluation on FF++ datasets. The best and second-best results are highlighted in bold and underline, respectively.

Methods	HQ (c23)		LQ (c40)	
	ACC	AUC	ACC	AUC
Two Branch [42]	–	86.59	–	98.70
Xception [21]	95.73	96.30	86.86	89.30
MADD [48]	96.37	98.97	86.95	87.26
F3Net [49]	97.52	98.10	90.43	93.30
SPSL [50]	91.50	95.32	81.57	82.82
Face X-ray [37]	–	87.35	–	61.60
FD-GAN [18]	98.12	**99.75**	91.60	**98.77**
**Ours(WAFF)**	**98.87**	98.42	**93.21**	96.57

**Table 5:** Cross-dataset AUC evaluation on public datasets. The best results are highlighted in bold.

Method	Input	Backbone	CDF	DFD	DFDC	DFDCp	FFIW
DSP-FWA [36]	Frame	ResNet50	69.30	-	-	-	-
Xception [21]	Frame	Xception	73.70	81.60	70.80	69.90	-
FRDM [38]	Frame	Xception	79.40	91.90	-	79.70	-
UIA-ViT [41]	Frame	ViT-B	82.41	94.68	69.80	75.80	58.74
SLADD [40]	Frame	Xception	79.70	-	76.05	-	-
CADDM [39]	Frame	EFNetB3	93.08	-	73.74	-	-
Face X-ray [37]	Frame	HRNet	79.50	95.40	-	80.92	-
FD-GAN [18]	Frame	Xception	84.2	97.1	77.2	-	-
Two-branch [42]	Video	LSTM	76.65	-	-	-	-
DAM [43]	Video	ResNet50	75.30	-	-	72.80	-
FInfer [44]	Video	GRU	70.60	-	-	70.39	69.46
**Ours(WAFF)**	Video	EFNetB3	**87.43**	**96.02**	**79.63**	**82.57**	**74.30**

**Table 6:** Lightweight diagnostic performance by FF++ generation type.

Generation Type	Videos	ACC	AUC	TP	FP	FN
Deepfakes	16	56.25	60.94	3	2	5
Face2Face	16	43.75	54.69	1	2	7
FaceShifter	16	50.00	46.88	2	2	6
FaceSwap	16	43.75	51.56	1	2	7
NeuralTextures	16	50.00	45.31	2	2	6

**Table 7:** Lightweight diagnostic FP/FN comparison of video-level fusion rules.

Rule	TP	FP	TN	FN	ACC	FPR	FNR
Fake-Frame Counting (FFC)	9	2	6	31	31.25	25.00	77.50
Confidence Averaging (CA)	5	1	7	35	25.00	12.50	87.50
Key-Frame Voting (KFV)	11	2	6	29	35.42	25.00	72.50
Ensemble Fusion	9	2	6	31	31.25	25.00	77.50

**Table 8:** Comparison of different backbone networks in terms of three factors.

Model	Top-1 Accuracy (%)	Params	FLOPs
ResNet-50	76.0	26 M	4.1 B
EfficientNet-B0	76.3	5.3 M	0.39 B
ResNet-152	77.8	60 M	11 B
Inception-v3	78.8	24 M	5.7 B
EfficientNet-B1	78.8	7.8 M	0.70 B
Xception	79.0	23 M	8.4 B
EfficientNet-B2	79.8	9.2 M	1.0 B
Inception-v4	80.0	48 M	13 B
EfficientNet-B3	81.1	12 M	1.8 B
SENet	82.7	146 M	42 B

**Table 9:** Comparison of decision rules on the mixed benchmark (1500 real + 1500 fake).

Decision Rule	ACC (%)	AUC (%)
Fake-Frame Counting (FFC)	83.5	86.2
Confidence Averaging (CA)	84.7	87.6
Key-Frame Voting (KFV)	85.4	88.1
Attention-Guided Fusion Decision (AGFD, ours)	89.6	92.8

**Table 10:** Comparison of weak and full supervision (AUC %, mean ± std over 5 runs).

Method	Supervision	FF++	Celeb-DF	DFDC-P
WAFF	Weak	98.87 ± 0.18	87.43 ± 0.32	82.57 ± 0.41
WAFF-FS	Full	99.29 ± 0.14	84.12 ± 0.45	76.84 ± 0.52

**Table 11:** Ablation study of frame sampling and preprocessing strategies. IT: inference time per video (ms); PV: prediction variance (lower is better).

Setting	IT (ms/Video)	PV
Frame Sampling Rate (aligned, scale = 1.3)		
Baseline (1 fps)	120	0.021
2 fps	210	0.020
4 fps	380	0.019
Face Alignment (1 fps, scale = 1.3)		
Aligned (baseline)	120	0.021
No alignment	95	0.047

## Data Availability

The data presented in this study are openly available in FaceForensics++ Dataset at https://www.kaggle.com/datasets/xdxd003/ff-c23 (accessed on 8 May 2026).
